# Bioactive Silk Revolution: Harnessing Curcuminoid Dye and Chitosan for Superior Antimicrobial Defence and UV Shielding

**DOI:** 10.3390/pharmaceutics16121510

**Published:** 2024-11-24

**Authors:** Khai Ly Do, Taswar Ahsan, Abdul Wahab, Muhammad Tayyab, Xinqi Yin, Nengjie Pan, Tao Huang, Asim Mushtaq, Miao Su

**Affiliations:** 1 School of Materials Science and Engineering, Zhejiang Sci-Tech University, Hangzhou 310018, China; 2 Shengzhou Innovation Research Institute, Zhejiang Sci-Tech University, Shaoxing 312451, China; 3 Institute of Plant Protection, Liaoning Academy of Agriculture Sciences, Shenyang 110161, China; 4 Institute of Materials Research, Tsinghua Shenzhen International Graduate School, Tsinghua University, Shenzhen 518055, China; 5 College of Textile Science and Engineering (International Silk Institute), Zhejiang Sci-Tech University, Hangzhou 310018, China; 6 Institute for Intelligent Bio/Chem Manufacturing (*i*BCM), Zhejiang University-Hangzhou Global Scientific and Technological Innovation Center, Hangzhou 311200, China

**Keywords:** curcuminoid, natural dye, antimicrobial, UV protective, functional silk

## Abstract

Background/Objectives: The use of natural colourants is gaining attention due to their biocompatibility and functional benefits. This study introduces a different approach using turmeric (*Curcuma longa* L.) dye extract combined with chitosan to significantly enhance the antibacterial and UV-shielding properties of silk. Methods: The turmeric dye’s chemical composition was analyzed using liquid chromatography mass spectrometry (LC-MS), UV–visible spectroscopy, and Fourier-transform infrared spectroscopy (FTIR). The dyed silk’s colourfastness was tested through rubbing, washing, and light exposure. Results: The chitosan-mordanted silk showed strong antibacterial activity against *Escherichia coli* (*E. coli*) and *Staphylococcus aureus* (*S. aureus*), as well as antifungal activity against *Aspergillus niger* (*A. niger*). It also demonstrated a high ultraviolet protection factor (UPF). For comparison, alum-mordant was used, and chitosan proved more effective. Beyond its use as a dye, turmeric is renowned for its medicinal properties. Its antioxidant, anticancer, and anti-inflammatory properties have been extensively researched, which are primarily linked to its curcuminoid compounds. Turmeric is used in traditional medication to treat digestive issues, arthritis, and skin diseases. Conclusions: This work underscores the innovative use of plant-based dye extracts and natural mordants like chitosan as a sustainable alternative to conventional metallic mordants, paving the way for the evolution of bioactive silk with improved functional properties.

## 1. Introduction

For centuries, plants and herbs have served as a vibrant treasure trove of natural colourants, with their hues being steeped in history and tradition. This practice persisted until the mid-nineteenth century when a British researcher introduced the synthetic dye named Mauveine (C.I. 50245). This innovation led to a shift towards synthetic dyes due to their lower costs, easy availability, and wide range of colours [1]. Today, the textile industry is one of the most hazardous industries, with textile effluents containing up to 45% dyes being discharged into the environment [2]. The production and utilization of over 10,000 types of synthetic colourants in the global market, particularly chemical and metal-based dyes in textile processes, adversely affect aquatic ecosystems, leading to an estimated 47% of the world’s population facing clean water scarcity by 2030. These synthetic dyes also pose serious health hazards to humans, including organ damage, DNA disruption, and cancer [3,4]. In response to these issues, plant-based dyes and biomass are being explored as eco-friendly alternatives. Many plant extracts provide a wide range of colours and help address health and environmental concerns due to their noncarcinogenic and biocompatible properties [5].

Plant-based colourants consist of numerous organic compounds with distinct chemical structures. These substances can be classified into five categories, including quinones, polyphenols, alkaloids, carotenoids, and porphyrins. Most anthraquinones, such as alizarin, emodin, purpurin, rubiadin, and morindone, are generated from the parental structure of 9,10-dioxoanthracene and create a range of red-to-brown shades [6]. In contrast, the anthocyanins in the polyphenol category, which differ from one another in terms of the position and quantity of benzene ring substitutes, yield red, yellow, blue, and violet hues based on their pH levels [5,7]. Carotenoids, which are differentiated by conjugated double bonds in their molecular structures, provide orange, red, and yellow colours and are commonly utilized for food colouration [8]. Alkaloid-based colourants predominantly comprise indigo and betalains, which provide blue and violet hues, respectively [9,10], whereas porphyrins are principally the Mg-tetrapyrrole molecules of chlorophylls, which produce blue and greenish pigments [5]. These colourants not only yield the hues, but also exhibit functional properties such as antimicrobial [11,12], antioxidant [13,14], anti-inflammatory [15], and UV protection [16] by virtue of the activities of their functional groups. Identifying the natural compounds present in plant-based dye extracts is of great importance for ensuring the authentication of herbs and plants, selecting the optimal extraction technology, choosing the appropriate dyeing method, and determining the functional qualities they possess [17]. Several techniques have been developed for natural compound identification, including UV-vis spectroscopy [18], FTIR [19], and Raman spectroscopy [20]. However, chromatographic analysis is regarded as the most sophisticated and reliable approach for identifying natural compounds. It involves monitoring the quantitative characteristics of analytes through high-resolution separation and establishing their chromatographic profiles [21,22,23].

**Figure 1:**
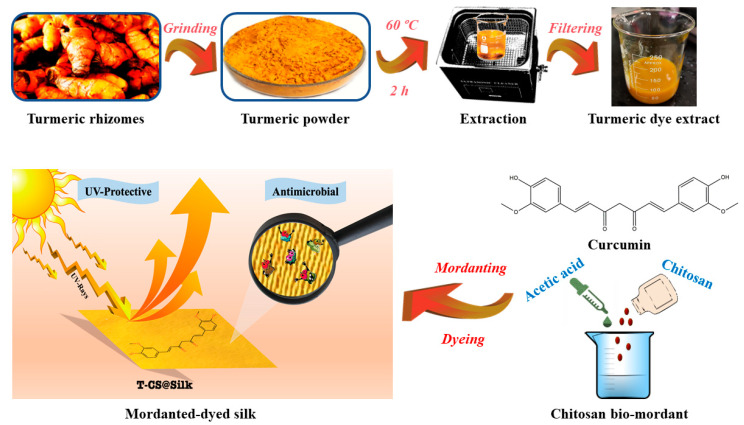
Schematic depiction of turmeric dye extraction and its applications on silk.

Curcuminoids, the naturally occurring polyphenolic molecules found in the rhizomes of turmeric (*Curcuma longa* L.) plants of the family Zingiberaceae, are highly valued in the market due to their wide range of applications as a food spice [24], colourant [25,26], and medicinal substance [27,28]. Curcumin can be regarded as a biocompatible compound when utilized at appropriate concentrations [29,30,31]. Its chemical structure facilitates interactions with microbial cell molecules, resulting in notable biological features, including its capacity to suppress the growth of various pathogenic colonies [32,33,34]. In addition, chitosan, an animal-based polysaccharide chemically comprising β(1→4)-linked D-glucosamine, has been effectively exploited as an antimicrobial agent in both its individual form and composite designs due to its biological activity that disrupts the structures and functions of microbial cell walls and membranes [35,36]. The role of chitosan as a bio-mordant in textile dyeing with natural colourants has also been recognized in a number of studies by virtue of its capability to generate robust dye complexes and adhere to targeted fibres [37,38,39]. The present research entails the utilization of a turmeric dye extract while employing chitosan to be a nature-based mordant to create silk substrates exhibiting an antimicrobial performance and UV protection. The chromatographic method was employed to confirm the existence of curcuminoid-based compounds in the dye extract derived from turmeric rhizomes. A chitosan pre-treatment followed by dyeing with turmeric extract was implemented on silk with the purpose of determining its antimicrobial activities against *Escherichia coli* (*E. coli*), *Staphylococcus aureus* (*S. aureus*), and *Aspergillus niger* (*A. niger*). This outcome was examined by comparing it with silk dyed without mordant and silk dyed with an alum mordant. The UV-protective behaviour of the silk substrates was assessed based on their measured UPF and radiation transmittance percentages. Figure 1 presents a schematic depiction of the experimental works. The main objective of this investigation is to promote the sustainable use of plant-based materials in the production of versatile textile products.

## 2. Materials and Methods

### 2.1. Materials

The turmeric rhizomes were purchased from a nearby agricultural farm (Sichuan, China). Acetic acid with a concentration of 99.7% (*v*/*v*), chitosan with a viscosity of 200–400 mPa.s and deacetylation degree of 80–90%, and potassium aluminum sulphate [K_2_SO_4_Al_2_(SO_4_)_3_·24H_2_O] were provided by Macklin Biochemical Co. Ltd. (Shanghai, China). These chemicals were in use for experiments at a high level of purity and precision.

Plain-woven white silk fabric, measuring 0.15 mm in thickness and weighing 51 g/m^2^, featuring a density of 135 warps and 97 wefts per inch, was provided by Find Silk Co., Ltd. (Hangzhou, China).

Grade-1 filter papers were purchased from Whatman (Maidstone, UK). These papers have a pore size of 11 μm, a diameter of 125 mm, a thickness of 180 μm, and a basic weight of 87 g/m^2^. Phosphate reference detergent B by European Colourfastness Establishment (ECE) was acquired from SDL Atlas (Shenzhen, China).

Moreover, *E. coli* (ATCC 8099) and *S. aureus* (ATCC 6538) were provided by Shanghai Amoy Strain Biotechnology Co., Ltd. (Shanghai, China).

### 2.2. Methods

#### 2.2.1. Extraction of Turmeric Dye

The dried turmeric rhizomes were properly peeled and pulverized using one ordinary food grinder. An aqueous solution of turmeric powder was prepared, controlling the material-to-liquor ratio to 1:5 (*w*/*v*), and allowing it to sit for 12 h at 25 °C. Following this, the mixture was kept for 120 min at 60 °C temperature, aided by ultrasonic waves (frequency: 45 kHz; power: 120 W) in one ultrasonic bath. Finally, the pure turmeric dye solution was acquired by employing grade-1 filter paper sheets.

Later, a fraction of the prepared dye solution was stored in 50 mL centrifuge tube and subjected to pre-freezing at −80 °C within 24 h. This was followed by a freeze-drying process using a Scientz-12N-A vacuum lyophilization instrument (Ningbo, China) at −47 °C for a duration of 72 h. The lyophilized material was stored for an FTIR test.

#### 2.2.2. Mordanting for Silk

A 1% (*v*/*v*) acetic acid solution was first made by adding 1 mL of acetic acid to 100 mL of water. Subsequently, 0.1 g of chitosan was introduced to the as-prepared acetic acid solution, with continuous stirring and the temperature maintained at 25 °C for 8 h. The silk was then immersed in this solution for 60 min at 60 °C, with a material-to-liquid ratio of 1:30 (*w*/*v*). Afterward, the treated silk was taken out and dried in an oven at 70 °C for 15 min.

Similarly, a mixture of K_2_SO_4_Al_2_(SO_4_)_3_·24H_2_O and distilled water was prepared with a material-to-liquid ratio of 1:25 (*w*/*v*), followed by stirring for 60 min at 25 °C. The silk was subsequently soaked in the alum solution for 60 min at 60 °C, while maintaining a material-to-liquid ratio of 1:30 (*w*/*v*). Finally, the treated silk was taken out and dried in an oven for 15 min at 70 °C.

#### 2.2.3. Dyeing for Silk

Pristine silk and the mordanted samples were separately immersed in the turmeric dye solution. The dyeing process was conducted in an electric dye bath for 60 min at 70 °C, with a fabric-to-liquid ratio of 1:40 (*w*/*v*). Subsequently, the fabrics were allowed to air dry gradually. The silk dyed without mordant was labelled T@Silk. The silk dyed with chitosan and alum mordants were designated as T-CS@Silk and T-Al@Silk, respectively. The pristine silk was preserved as the control sample.

### 2.3. Characterization Techniques

#### 2.3.1. Liquid Chromatography—Mass Spectrometry (LC-MS)

An LC-MS analysis was carried out using an Agilent 6470 triple-quadrupole tandem mass-spectrometer (Agilent, Beijing, China) to identify the main components of the turmeric dye extract, based on a previously published study [40]. The analysis was conducted with a 17,000 Da/sec maximum scanning rate over a 5–2900 *m*/*z* mass range. An Eclipse Plus C18 RRHD column (1.8 µm, 2.1 × 50 mm) was utilized, which was maintained at a temperature of 30 °C. In addition, the mobile phase consisted of (A) 100% water and (B) a 1:1 (*v*/*v*) mixture of methanol and acetonitrile. The gradient programme was as follows: 95% A and 5% B for the first minute, then 95% B from 1 to 8 min, and 95% B maintained from 8 to 11 min. The injection volume was 0.3 µL, with a flow rate of 0.3 mL/min. The analysis was conducted in positive-ionization mode using an electrospray ionization (ESI) source, controlled by Mass Hunter software (version 10.1) intended for qualitative analysis. The system operated with a capillary voltage of 3500 V, a gas flow of 5 L/min, a nebulizer pressure of 35 psi, and a gas temperature of 300 °C.

#### 2.3.2. Silk Characterization

An FTIR spectrometer (Nicolet iS20, Thermo Fisher Scientific, Waltham, MA, USA) was utilized to analyze the FTIR spectra of the lyophilized turmeric extract as well as the silk samples. Each sample underwent thirty-two scans in total, utilizing the attenuated total reflectance (ATR) mode for the silk samples and the KBr pellet method for the turmeric extract. The scans had a spectral resolution of 4 cm^−1^ and covered a wavenumber range of 500 to 4000 cm^−1^. Furthermore, a double-beam UV-vis spectrophotometer (MAPADA P7, Shanghai Mapada Instruments Co. Ltd., Shanghai, China) was used to record the UV-vis spectrum of the turmeric extract, covering a spectral range of 200 to 900 nm.

Additionally, the XRD profiles of the studied silk samples were analyzed by utilizing one D2 Phaser XRD equipment (Bruker, Karlsruhe, Germany). The experiment utilized Cu Kα radiation at a scan rate of 1 s, with an applied voltage of 30 kV and a 10 mA current. The step size for each measurement was 0.02 degrees. The inter-atomic distance (d-spacing) was calculated by following the Bragg’s equation:

*d*-spacing = λ/2 sinθ

where λ represents the X-ray wavelength of 1.54 Å and 2θ stands for the X-ray scattering angle ranging from 5 to 60 degrees.

Also, the silk samples’ surface morphology and elemental mapping were captured by using a Sigma 300 FE-SEM (Zeiss, Oberkochen, Germany) at 2.5 kV.

#### 2.3.3. Colouring Property

A Datacolor SF600 Plus colorimeter (Datacolor, Suzhou, China) was operated with a D65 illuminant, maintaining an observation angle of 10° in a wavelength ranging from 360 to 700 nm, to ascertain the colour strength (K/S) of the dyed silk by averaging three measurements from each sample. The L*, a*, b* characteristics of the CIE colour space were concurrently documented to assess the colour coordinates. The L* value presents the degree of lightness, ranging from black to white. The a* value varies from negative (green) to positive (red), whereas b* varies from negative (blue) to positive (yellow).

#### 2.3.4. Air Permeability

The air permeability testing of the dyed silk samples was performed with an air pressure of 100 Pa through a metallic nozzle, adhering to the American Society for Testing and Materials standard (ASTM D737-18) [41], by using a YG461E tester (Ningbo Textile Instrument Factory, Ningbo, China).

### 2.4. Colour Fastness Analysis

The fastness of the dyed silk was assessed through washing, rubbing, and light exposure tests. The dyed silk underwent a 30 min wash at 40 °C in M228 Rotawash equipment (SDL Atlas, Shenzhen, China) with ECE detergent (4 g/L) and ten metallic spheres, following the standard of the International Standard Organization (ISO) as ISO 105-C06:2010 [42] for washing fastness examination. Dry rubbing was conducted using an Atlas crock-metre (Shenzhen, China) with 10 turns, as per the ISO 105-X12:2016 [43]. A wet rubbing analysis followed, with the silk being moistened and rubbed similarly to dry rubbing. Light fastness was tested under a xenon arc lamp (Lixian Instrument Scientific Co., Ltd., Dongguan, China), simulating natural daylight conditions and adhering to ISO 105-B02:2013 [44].

The fastness findings were evaluated using a grayscale in accordance with ISO 105-A02:1993 [45] for determining colour change, with grades from 1 (notable change) to 5 (no discernible change).

A quantitative investigation was implemented to examine the colour difference (∆E*) of the dyed fabrics pre- and post-washing using the equation:

$$∆E*=\sqrt{{\left(∆L^{*}\right)}^{2}+{\left(∆a^{*}\right)}^{2}+{\left(∆b^{*}\right)}^{2}}$$

in which $∆L^{*}$, $∆a^{*}$, and $∆b^{*}$ present the differences in the colour coordinates of the unwashed and washed silk. The ∆E* ratings were assessed according to Zachary Schuessler’s theory, wherein a ∆E* less than 1 reflects that the colour difference cannot be recognized by human eyes, a ∆E* between 1 and 2 signifies that the colour difference is discernible upon close inspection, a ∆E* from 2 to 10 denotes a colour difference noticeable at a glance, a ∆E* ranging from 11 to 49 reflects colours with slight similarity, and a ∆E* exceeding 49 indicates a complete contrast between the colours [46].

### 2.5. Investigation of Functional Properties of Silk

#### 2.5.1. Antibacterial Test

The antibacterial activity of silk against *E. coli* and *S. aureus* strains was assessed in vitro using a methodology described by Hou and co-workers [47]. Initially, the silk was cut into precise 1 cm circular discs, followed by a sterilization step using UV irradiation (15 W) within 30 min. In addition to that, both types of bacteria were separately cultured in micro-vials containing LB broth (10 mL), with an incubation temperature maintained at 37 °C. Subsequently, the sterilized silk specimens were co-incubated in bacterial suspensions that had been previously diluted with phosphate-buffered saline (PBS) to a concentration of 10^6^ CFU/mL. After 24 h of incubation, 100 μL of each bacterial suspension was assigned to each well of the 96-well plate and subjected to a measurement of optical density at 600 nm (OD_600_) using a SpectraMax M5 reader (Marshall Scientific, New Hampshire, United States). The experiments were conducted three times for each sample to acquire the average values.

The percentages of bacterial inhibition upon *E. coli* and *S. aureus* were calculated by using one formula as follows:

Inhibition percentage (%) = (Blank OD_600_ − Silk OD_600_/Blank OD_600_) × 100%

For the determination of the bacteriostatic circle, the prepared 100 μL (10^6^ CFU/mL) samples of *E. coli* and *S. aureus* were separately dropped onto LB solid bacterial medium, which was consistently coated with the bacterial solution with a glass spreader. Circular silk discs were strategically positioned on a solid culture medium and incubated at 37 °C for 24 h. Following incubation, the diameter of the inhibition zone was meticulously measured using a calibrated scale, revealing the antimicrobial efficacy.

#### 2.5.2. Antifungal Test

The silk samples were subjected to an intense examination of their antifungal properties, focusing on their ability to combat white mould (*A. niger*). The pathogenic strain of white mould was taken from the Institute of Plant Protection, Liaoning Academy of Agricultural Science, China. This aggressive fungus was cultivated in potato dextrose agar (PDA). Firstly, turmeric dye extracts with concentrations of 0 µL, 25 µL, 50 µL, and 100 µL were individually used to examine the growth of fungi. The next step was to hydrate the silk, ensuring it was prepared to interact fully with the fungal spores (10^7^ spores/mL). Once properly conditioned, the silk samples were exposed to a spore suspension of the fungus. The test was meticulously carried out with an incubation period of 15 days at 28 °C. Following the incubation, the detached leaf method was employed, a technique renowned for its precision in isolating and measuring fungal activity. The colonial diameter of the mould colonies was meticulously measured, offering critical insights into the viability and growth of the spores. This allowed for a detailed understanding of how the varying turmeric concentrations influenced the fungal growth patterns. The results were calculated and averaged from the three repeated experiments detailed by Bellotti and colleagues [48]. This ensured that the results were not only qualitative but also robust, allowing for an objective evaluation of the silk’s antifungal efficacy against white mould.

#### 2.5.3. UV Protection Test

Using a UV-2000F Textiles UV Factor Tester (Labsphere, North Sutton, NH, USA), the UPF and UV transmittance rates of the silk specimens were evaluated following the guidelines of the American Association of Textile Chemists and Colorists (AATCC) as AATCC 183-2000 [49]. The UV-A and UV-B transmittance rates, along with the UPF, were subsequently calculated using the provided formulas:

$$\mathrm{UPF}=\int_{280}^{400}E\left(\lambda \right)S\left(\lambda \right)d/\int_{280}^{400}E\left(\lambda \right)S\left(\lambda \right)T\left(\lambda \right)d$$

$$\mathrm{UV}-A\;\mathrm{transmittance}\;\mathrm{rate}=100\int_{320}^{400}T\left(\lambda \right)d\lambda /\int_{320}^{400}d\lambda \;(\%)$$

$$\mathrm{UV}-B\;\mathrm{transmittance}\;\mathrm{rate}=100\;\int_{280}^{320}T\left(\lambda \right)d\lambda /\int_{280}^{320}d\lambda \;(\%)$$

In this context, S(λ) denotes the spectral irradiance, T(λ) represents the average spectral transmittance of the fabric at wavelength λ, E(λ) represents the relative erythema spectral effectiveness, and dλ signifies the bandwidth.

UPF values below 15 are considered poor, values from 15 to 24 are considered good, those in the range of 25 to 39 are considered very good, and values above 39 are regarded as excellent.

Moreover, the UV blocking rate was calculated as follows:

UV blocking rate = 100 − UV-transmittance rate (%)

## 3. Results and Discussion

### 3.1. Characterizations of Turmeric Dye Extract

**Figure 2:**
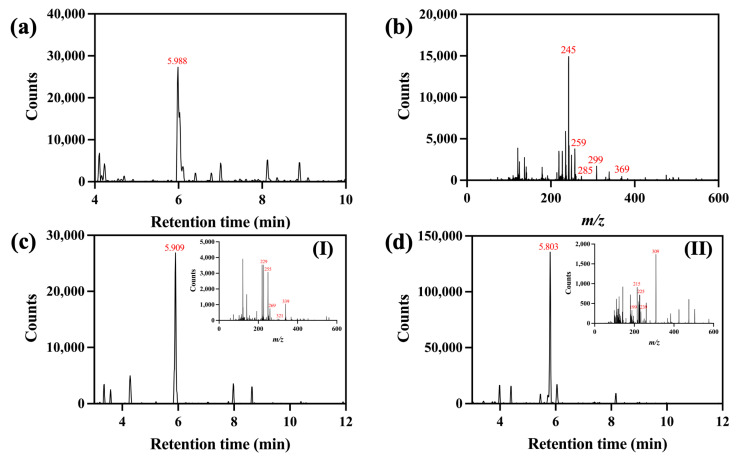
LC-MS analysis results for turmeric dye extract: (**a**,**b**) chromatogram and mass spectrum of the peak at 5.988 min (curcumin), respectively, and (**c**) chromatogram; inset I: mass spectrum of the peak at 5.909 min (demethoxycurcumin), and (**d**) chromatogram; inset II: mass spectrum of the peak at 5.803 min (bisdemethoxycurcumin).

The chromatographic data for the turmeric dye extract are displayed in Figure 2. According to Figure 2a, one prominent peak was found at a retention time of 5.988 min with its molecular ion ([M+H]^+^ = *m*/*z* 369). Its fragment ions were possibly generated due to the loss of one diketocyclopropane ([M+H–C_3_H_2_O_2_]^+^ = *m*/*z* 299), the loss of one 1-hydroxy-3-ketocyclobutene (M+H–C_4_H_4_O_2_)^+^ = *m*/*z* 285), the loss of one 1-hydroxy-5-ketocyclo-1,3-hexadiene (M+H–C_6_H_6_O_2_)^+^ = *m*/*z* 259), and the loss of one aryl group followed by one hydrogen atom (M+H–C_7_H_7_O_2_–H)^+^ = *m*/*z* 245) (Figure 2b). This analytical result suggested the presence of curcumin in the dye extract. The chromatogram detected at a retention time of 5.909 min (Figure 2c) with a molecular ion ([M+H]^+^ = *m*/*z* 339) and fragment ions presumably created by the loss of one water molecule (M+H–H_2_O)^+^ = *m*/*z* 321), the deduction of one diketocyclopropane ([M+H–C_3_H_2_O_2_]^+^ = *m*/*z* 269), the loss of one 1-hydroxy-3-ketocyclobutene (M+H–C_4_H_4_O_2_)^+^ = *m*/*z* 255), and the subtraction of one 1-hydroxy-5-ketocyclo-1,3-hexadiene (M+H–C_6_H_6_O_2_)^+^ = *m*/*z* 229) (inset I) indicated the presence of demethoxycurcumin. In a related manner, one molecular ion ([M+H]^+^ at *m*/*z* 309) was recorded at a retention time of 5.803 min (Figure 2d) alongside its fragment ions, probably produced by the removal of one diketocyclopropane, one 1-hydroxy-3-ketocyclobutene, and one 1-hydroxy-5-ketocyclo-1,3-hexadiene at *m*/*z* 239, 225, and 199, respectively. Another fragment, *m*/*z* 215, was possibly formed by the elimination of one hydrogen and one aryl (inset II). This revealed that the bisdemethoxycurcumin component was available [50,51]. The LC-MS results confirmed the existence of curcuminoid-based compounds in the turmeric extract, which would be responsible for providing its yellow shade and functional activities on fibrous substrates.

**Figure 3:**
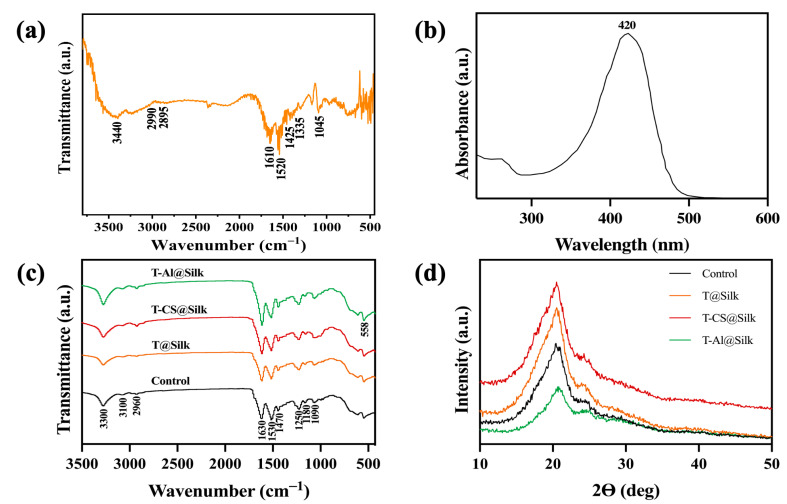
(**a**) FTIR spectrum of turmeric dye extract, (**b**) UV-vis spectrum of turmeric dye extract, (**c**) FTIR spectra of silk samples, and (**d**) XRD patterns of silk samples.

The FTIR spectrum of the turmeric dye extract, shown in Figure 3a, unveils a rich array of absorption bands that provide insight into its molecular structure. A striking absorption at 3440 cm^−1^ reveals the O–H stretch of phenolic groups, signalling the presence of hydroxyl functionality. The peaks at 2990 and 2895 cm^−1^ are attributed to the characteristic stretches of methyl (–CH₃) and methylene (–CH₂) groups, respectively. A broad band at 1610 cm^−1^ marks the C=O stretch, indicative of conjugated carbonyl groups, while a sharp peak at 1520 cm^−1^ highlights the aromatic C=C stretching vibration, confirming the presence of aromatic rings. Further, the bands at 1425 and 1335 cm^−1^ correspond to bending vibrations of CH₂ and CH₃ groups, respectively, with the peak at 1045 cm^−1^ being assigned to the C–OH stretching, offering a detailed molecular fingerprint of the extract [52,53,54].

The UV-vis spectrum in Figure 3b indicated that the turmeric dye extract presented a visible absorption band in a wavelength range (360–470 nm) with a maximum absorption peak located at 420 nm. It was claimed that the electron excitation generated by the π-π* electronic transition could account for this absorption band in the turmeric extract in the visible region [55]. Furthermore, the maximum absorption peak at 420 nm probably owed to the diarylheptanoid group of curcumin [56].

### 3.2. Silk Characterizations

The FTIR analysis serves as a powerful tool to uncover the functional groups embedded within the structure of silk fibres. As illustrated in Figure 3c, a series of distinct absorption peaks reveal the molecular composition of the silk. The peaks at 3300 cm^−1^ and 3100 cm^−1^ correspond to the O–H and N–H stretching vibrations, respectively, highlighting the presence of hydroxyl and amine groups. The prominent peak at 2960 cm^−1^ is attributed to the antisymmetric C–H stretching, further confirming the organic nature of the silk. The signature peaks for Amide I and Amide II are observed at 1630 cm^−1^ and 1530 cm^−1^, indicative of the protein’s secondary structure. Additionally, peaks at 1470 cm^−1^, 1250 cm^−1^, 1180 cm^−1^, and 1090 cm^−1^ are assigned to C–H bending, Amide III stretching, C–N stretching in tyrosine, and C–C stretching, respectively, collectively revealing the intricate molecular architecture of the silk fibres [57,58]. On the other hand, the varying absorption of the peaks at 3300 cm^−1^ in the dyed silk samples reflected the alteration in their phenol hydroxyl groups [59]. In addition, a small increase in the intensity of the peak at 2960 cm^−1^ was recognized in the FTIR spectrum of the T-CS@Silk sample, which might be attributed to the presence of the –CH_2_ group in chitosan [60]. The high-intensity peak observed at the wavenumber of 558 cm^−1^ in the FTIR spectrum of the T-Al@Silk sample was likely owing to the appearance of the Al–O bond [61].

The XRD analysis was implemented in order to monitor the alteration in the secondary structure of silk fibres before and after dyeing with turmeric dye extract. As exhibited in Figure 3d, the pristine silk has two primary crystalline peaks. One peak, corresponding to the (002) plane, was recorded at an angle of 2θ = 24.3°, while another one presenting along the (210) plane was measured at 2θ = 20.2°. In addition, the *d*-spacing values for the (210) and (002) planes were 0.44 and 0.37 nm, individually. This outcome aligned with the reported *ß*-sheet crystalline conformation of silk fibres [62,63,64]. It could be recognized that the peak positions in the T@Silk, T-CS@Silk, and T-Al@Silk samples were unchanged. This revealed that the process of mordanting and dyeing transpired in the amorphous areas of silk without disturbing its crystal structure [65].

**Figure 4:**
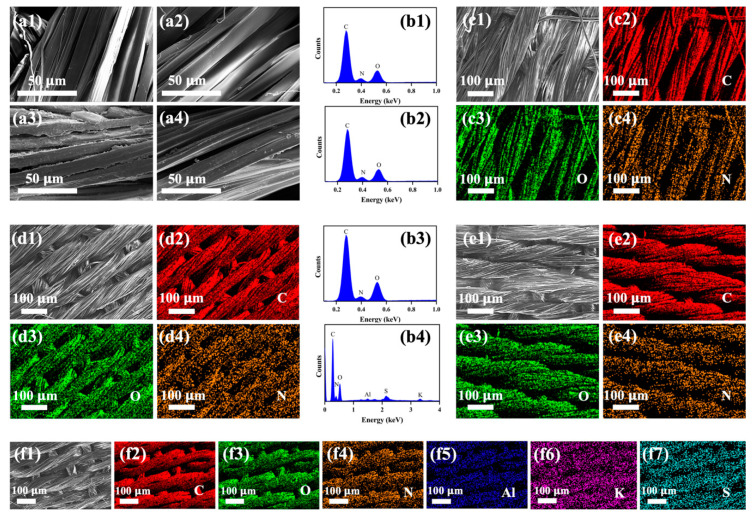
SEM images of silk samples: (**a1**) pristine silk, (**a2**) T@Silk, (**a3**) T-CS@Silk, (**a4**) T-Al@Silk; EDS spectra of silk samples: (**b1**) pristine silk, (**b2**) T@Silk, (**b3**) T-CS@Silk, (**b4**) T-Al@Silk; (**c1**–**c4**) elemental mappings of pristine silk; (**d1**–**d4**) elemental mappings of T@Silk; (**e1**–**e4**) elemental mappings of T-CS@Silk; (**f1**–**f7**) elemental mappings of T-Al@Silk.

The morphological determination of silk samples is crucial for assessing the effect of dye complexes on the surface structure of the fibres. The SEM micrographs of the silk samples under investigation are displayed in Figure 4. It was shown that the plain silk had a distinctly clean and smooth surface (Figure 4(a1)), and the silk dyed directly with the turmeric extract did not cause any deformity in the silk fibres but instead formed a slender coating layer (Figure 4(a2)). The silk sample pre-treated with chitosan and dyed with turmeric extract exhibited a noticeable increase in surface roughness, probably owing to the existence of the concentrated dye materials (Figure 4(a3)). On the other hand, the silk that underwent pre-treatment with alum and was dyed showed a moderate level of roughness with fewer clusters on the surface (Figure 4(a4)).

The EDS and elemental mappings detected the existence of several elements on the surface of silk fibres. Figure 4(b1–b3) reveals that the undyed silk, T@Silk, and T-CS@Silk samples contain carbon (C), oxygen (O), and nitrogen (N), which are the typical elements found in silk fibres [64,66]. The T-Al@Silk sample showed the presence of alum (Al), potassium (K), and sulphur (S), which are the elements of alum mordant, in addition to the ordinary elements of silk (Figure 4(b4)). Along with that, the elemental mappings of the silk samples helped validate the proper distribution of the aforementioned elements on silk fibres (Figure 4(c1–c4,d1–d4,e1–e4,f1–f7)).

### 3.3. Colouration and Fastness of Silk

Table 1 displays the colouring, fastness, and air permeability properties of the dyed silk. The experiment revealed that using turmeric dye extract for direct dyeing produced a pale-yellow hue on silk fabric, whereas dyeing in the presence of chitosan resulted in an orange-yellow shade of silk and dyeing with alum mordant significantly darkened the yellow shade. In addition to that, the T-CS@Silk sample had a higher K/S value (19.70) compared to those of the T@Silk sample (12.82) and T-Al@Silk sample (13.65). The lower L* of the T-CS@Silk sample (64.34) and T-Al@Silk sample (65.67) compared to the T@Silk (75.48) also reflected that the mordants reduced the lightness of the dyed fabric. Furthermore, a* and b* of all samples were acquired with positive (+) values, indicating that the shades corresponded to the yellow and red regions of the CIE colour space.

The T-CS@Silk exhibited a moderate-to-good fastness, specifically achieving scores of 4–5 for the light and washing testing, and 3–4 for the wet and dry rubbing. This demonstrates that chitosan effectively bonded the dye molecules to the silk fabric. This outcome was superior to that of the T@Silk, as this sample achieved scores of 2–3 in the rubbing and washing tests and 3–4 in the light fastness test. The T-Al@Silk did not earn great scores in terms of its fastness against washing, rubbing, and light, but it was maintained at a moderate level (3–4). In addition, only the T-CS@Silk exhibited a ∆E* value below 2, signifying that the colour difference resulting from washing could only be discerned under meticulous observation. Moreover, it was observed that the air-permeability of the T-CS@Silk (495.41 mm/s) was marginally lower in comparison to that of the T@Silk (554.91 mm/s) and T-Al@Silk (514.24 mm/s). This result suggests that the chitosan bio-mordant did not have a significant impact on the breathability of silk.

**Figure 5:**
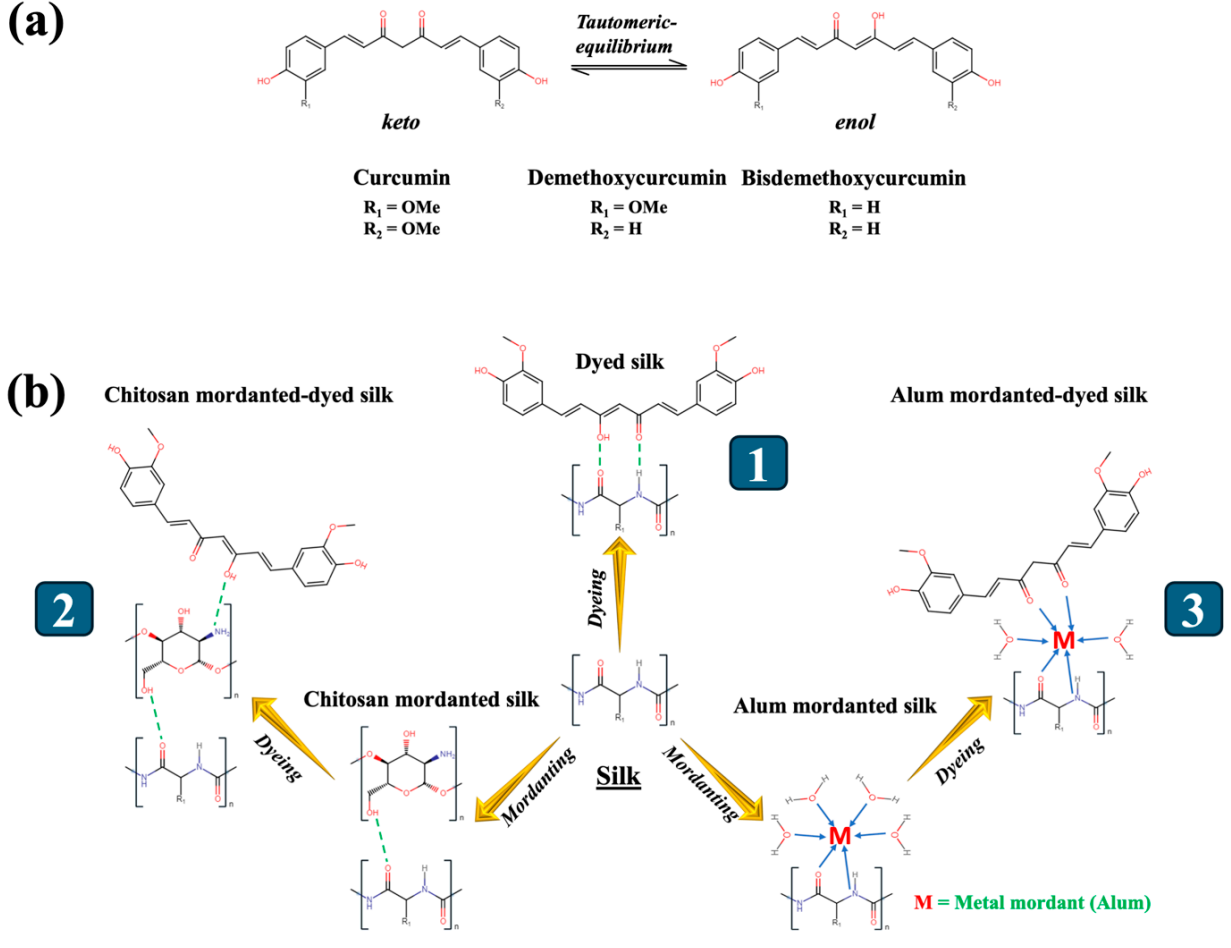
(**a**) Chemical formulae of curcuminoids in keto and enol forms; (**b**) proposed schemes of 1. dye-silk complex; 2. dye–chitosan-silk complex; and 3. dye–metal–silk complex.

The dyed silk samples exhibited vibrant colours and durable quality, indicating that the dye complexes effectively attached to the silk fibres, resulting in the desired shades and controlling the discolouration caused by water washing, exposure to light, and rubbing. The curcuminoid-based dye components in keto and enol forms (Figure 5a) were plausibly bonded to silk fibres by the bonds formed by the dye molecules with the carbonyl (>C=O) and the amino (–NH_2_) groups in silk (Figure 5(b1)) [67]. Nevertheless, the low washing-fastness ratings reflected the fragility and susceptibility to breaking of hydrogen bonds. Furthermore, the hydrophobic nature of curcuminoid molecules causes them to primarily aggregate in the dye solution and on the surface of silk fibres, rather than within the fibre pores. This ultimately led to a low level of resistance to rubbing [68]. Furthermore, turmeric dye molecules might not establish substantial aggregates inside silk fibres, which might inadequately regulate the passage of light energy from the fabric surface to the internal structure, thus the light fastness of the directly dyed sample was acquired only at a modest level [69].

When the silk sample was dyed in the presence of chitosan, it is most likely that ionic bonds were generated between the amino groups in chitosan and the –OH groups of curcuminoids [70]. Additionally, a chitosan molecule was plausibly attached to the silk structure through hydrogen bonds established between the oxygen atom of the C=O group in silk and the hydrogen atom from the –OH in chitosan (Figure 5(b2)) [71]. The dye–chitosan complex probably enhanced the capacity of the dye to bind to the silk fibres, leading to a greater K/S value and fastness for the T-CS@Silk compared to that of the simply dyed silk. Further, the high rating of the light fastness observed for the silk dyed in the presence of chitosan was probably credited to the ability of this bio-mordant to increase the concentration of dye complexes on silk. This ultimately led to a noteworthy decrease in the transmission of light energy from the surface of the silk to its inner structure [72]. In addition to that, curcuminoids have been reported to have the ability to create chelates with metallic mordants. With regard to the T-Al@Silk, the chelates were potentially produced by the metal atom and the ligand created by the C=O groups of the dye molecules [73,74]. Along with that, the metal ion interacted with the carbonyl and amino group of silk, thus binding the dye complex to the fibres (Figure 5(b3)) [21]. It has been hypothesized that metal-based mordants have the ability to augment the durability of natural-dyed silk fabrics by creating dye complexes with bigger molecular sizes and reduced water solubility, thus rendering them more resistant to removal from silk [75].

### 3.4. Functional Performances of Silk

#### 3.4.1. Antibacterial Activity

**Figure 6:**
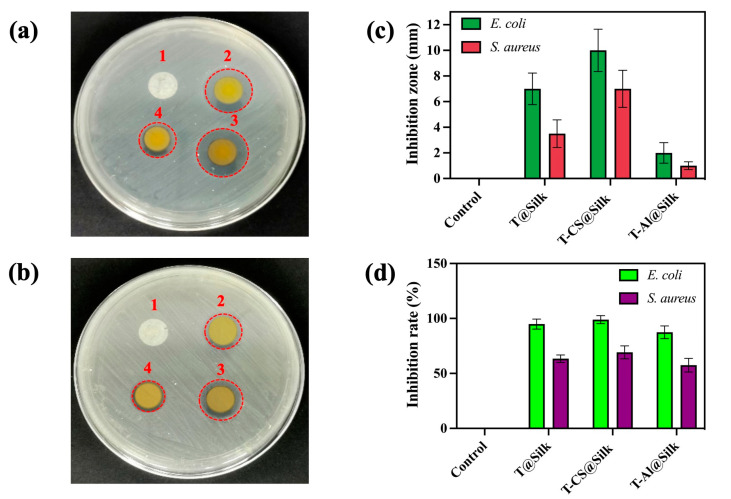
Antibacterial study of silk samples against *E. coli* and *S. aureus*: (**a**) antibacterial circles of silk against *E. coli*, (**b**) antibacterial circles of silk against *S. aureus* (1: Control, 2: T@Silk, 3: T-CS@Silk, 4: T-Al@Silk), (**c**) measured inhibition zones, (**d**) bacterial inhibition percentages (%).

Figure 6 provides a description of the antibacterial effects of the silk samples being studied against *E. coli* and *S. aureus* strains. According to Figure 6a,b, the control showed no bacterial inhibition against both types of bacteria, whereas T-CS@Silk displayed larger inhibition circles compared to the other samples. This finding corresponded with the measured inhibition zones, in which the zones of T-CS@Silk were 10 and 7 mm for *E. coli* and *S. aureus*, individually, and larger than those of the T@Silk and T-Al@Silk samples (Figure 6c). The measured OD_600_ values of the control were 1.14 and 1.06 when exposed to *E. coli* and *S. aureus* individually. The OD_600_ values of the coloured silk specimens exhibited a substantial drop. In particular, the silk that was dyed directly had an OD_600_ of 0.19 and 0.42 against *E. coli* and *S. aureus* individually. The lowest OD_600_ values among the studied samples were acquired in the T-CS@Silk (OD_600_ = 0.15 for *E. coli* and 0.36 for *S. aureus*). Despite the T-Al@Silk displaying lower bacterial concentrations compared to the control, its OD_600_ values (0.28 and 0.48 for *E. coli* and *S. aureus*, respectively) were higher than those of the silk dyed without mordant. Based on the results of OD_600_ measurement, bacterial inhibition percentages were calculated and reported. Figure 6d reflects that the control hardly shows any inhibition against both species of bacteria, while the directly dyed silk has remarkable antibacterial inhibition percentages of 94% and 63% against *E. coli* and *S. aureus*, respectively. The most outstanding inhibition percentages among these samples were in the T-CS@Silk (98% for *E. coli* and 69% for *S. aureus*). Nevertheless, the T-Al@Silk had inhibition percentages of 87% against *E. coli* and 57% against *S. aureus*, which were noticeably lower compared to the inhibition percentages of the T@Silk and T-CS@Silk.

The results indicated that the plain silk exhibited minimal antibacterial behaviour against both bacterial strains. When it was simply dyed with the turmeric extract, the antibacterial performance was somewhat improved by virtue of the antibacterial property of the dye. It was reported that curcumin has the ability to impede the growth, creation of biofilm, and division of bacterial cells. The methoxy groups attached in the benzene rings of curcumin hindered the function of sortase A by chemically interacting with GLN-172, LEU-169, and VAL-168 sites of *S. aureus*. Curcumin also suppressed the creation of QS-dependent components such as the motility, exopolysaccharide synthesis, and alginate creation of *E. coli* [76,77]. Furthermore, the demethoxycurcumin component caused damage to the transpeptidase enzyme, resulting in the destruction of glycopeptide production in the bacterial cell walls [78]. In an era where numerous synthetic nanomaterials have been fabricated for antimicrobial research [79,80,81], a plant-derived extract like turmeric remains a viable choice for combating pathogens by virtue of its potent antibacterial behaviour.

The utilization of both the bio-mordant and turmeric extract led to an outstanding antibacterial performance of the T-CS@Silk sample. It has been proposed that chitosan exerted its antibacterial function by binding to bacterial cell surfaces. This caused an elevation in the permeability of lipid cell membranes and the leakage of vital substances from bacterial cells, finally leading to cell demise [82]. The superior antibacterial efficacy of chitosan against *E. coli* compared to *S. aureus* might be due to the different compositions of Gram-negative and Gram-positive bacteria. The membranes of *E. coli* contain lipopolysaccharide molecules that have negatively charged phosphorylated groups. These groups readily interact with cationic chitosan, resulting in a notable increase in membrane permeability. The peptidoglycan layers of *S. aureus* include the teichoic acids, which are also negatively charged because of their phosphate groups. However, the inhibition of teichoic acid production in *S. aureus* confers resistance to chitosan in this bacterial strain [83,84]. All of this probably increased the vulnerability of *E. coli* to chitosan, as opposed to *S. aureus*. In addition, the use of alum mordant supported the enhancement of antibacterial effectiveness of the T-Al@Silk sample by demonstrating its toxicity towards pathogens in both its metallic compound and free form. Alum might form a bond with bacterial proteins and generate reactive oxygen species, which damaged the bacterial structures [85,86]. Nevertheless, it was likely that metal ions formed chemical bonds with the functional groups of the dye molecules, resulting in a reduced interaction between the dye and bacterial molecules [87].

#### 3.4.2. Antifungal Activity

**Figure 7:**
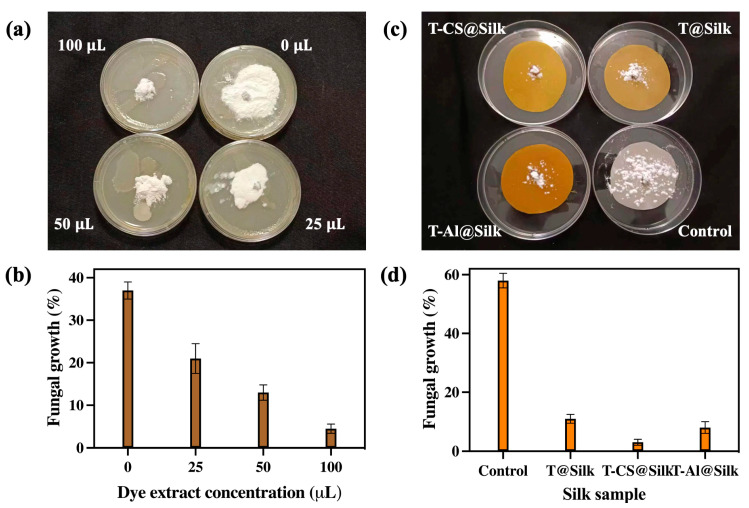
Antifungal study of turmeric dye extract and silk samples against white mould: (**a**) effect of variable dye extract concentration on fungal growth, (**b**) fungal growth percentages by variable dye extract concentrations, (**c**) fungal growth on silk samples, and (**d**) fungal growth percentages on silk samples.

Figure 7 displays the antifungal action of turmeric dye extract and silk samples against white mould. Figure 7a showcases that the fungal growth was considerably suppressed with a low concentration (25 μL) of the dye extract as opposed to the discernible proliferation of fungus in the absence of dye extract (0 μL). The suppression of fungal growth was further amplified upon increasing this concentration to 50 μL and 100 μL. A fungal growth rate of 37% was recorded in the absence of dye extract (0 uL), while a low concentration of dye (25 μL) resulted in 21% growth. This percentage was reduced to 13% and 4.5% using 50 μL and 100 μL dye extract, respectively (Figure 7b). Regarding the silk samples, Figure 7c and d show that the control had a dominating fungal colony and a high growth percentage of 58%, demonstrating the weak antifungal property of silk fibres. The T-CS@Silk sample exhibited the most remarkable fungal suppression with only 3% fungal growth, which was significantly lower than that of the T@Silk (11%) and T-Al@Silk (8%) samples.

It has been established that the primary mechanism by which turmeric extract exhibited its antifungal effect on white mould was its ability to destroy the fungal plasma membrane. It most likely stopped the ergosterol biosynthesis, which caused the fungal cell to stop functioning. Turmeric extract might also have prevented the development of mitochondrial ATP synthase, which would have decreased the intracellular ATP level, disturbed regular metabolic process, and ultimately resulted in cell death. The suppression of the plasma membrane caused by the disruption of mitochondrial ATP synthase also led to the destruction of the permeability and structure of the fungal cell membrane. In addition, turmeric extract was able to interfere with the tricarboxylic acid (TCA) cycle of fungal cells by inhibiting its major catalyzing enzymes, including malate dehydrogenase (MDH) and succinate dehydrogenase (SDH). This phenomenon prevented the process of energy metabolism in fungi [88]. The most outstanding antifungal performance of the T-CS@Silk among all the samples was owing to the combined action of turmeric extract and chitosan against white mould. Chitosan has been shown to rupture cell membranes, cause cytoplasm leakage, and interact with nucleic acids to modify genetic information [89].

#### 3.4.3. UV-Protective Activity

The UPF values, UV radiation transmittance, and blocking percentages quantify the UV protective ability of silk fabrics. Table 2 displayed the UPF of just 9 for the pristine silk. This sample exhibited UV radiation transmittance percentages of 19.72% for UV-A and 5.64% for UV-B. All of this reflected the inadequate UV-protective properties of plain silk. UPF 50^+^ was acquired in the dyed silk samples, demonstrating that the dyeing process greatly enhanced the UV-shielding capability of the silk fabric. The T-CS@Silk sample had the lowest transmittance percentages of UV radiation among the samples studied, with 0.73% for UV-A and 0.68% for UV-B. Its UV blocking percentages for UV-A and UV-B were 99.27% and 99.22%, individually.

The findings indicated that chitosan, instead of alum mordant, significantly improved the dye’s adherence due to the coordination-complexes among dye molecules and silk fibres. Increased concentration of these complexes resulted in better UV protection, as more energy was dissipated through the functional molecules of the dye, thereby maximizing radiation absorption.

## 4. Conclusions and Perspectives

This study evaluated the effectiveness of turmeric rhizome dye extract combined with chitosan in producing functional silk with antimicrobial and sun-protective properties. Chromatographic analysis identified curcuminoids (curcumin, demethoxycurcumin, and bisdemethoxycurcumin) in the dye extract. The dyeing process had minimal impact on the silk’s structure, aside from slight changes in fibre morphology. Silk pre-treated with chitosan and dyed with turmeric extract demonstrated a good fastness and impressive antimicrobial activity, inhibiting 98% of E. coli and 69% of S. aureus, while exhibiting strong antifungal activity, allowing only 3% fungal growth. The treated silk also offered excellent sun protection, achieving a UPF rating of 50^+^.

The study highlighted that using chitosan as a mordant enhanced the functional properties of silk more effectively than traditional alum. Given the growing need to minimize hazardous chemicals, plant-based dyes and biomass present eco-friendly and versatile alternatives, offering affordability, biocompatibility, and a wide array of bioactive compounds. Turmeric extract, known for its medicinal, cosmeceutical, and nutritional potential, shows promise for scaling up in textile applications, especially in the development of medical textiles due to its potent antimicrobial properties.

### Future Perspectives

In the realm of pharmaceutics, the findings of this study suggest promising avenues for the use of turmeric dye in developing bioactive fabrics for wound dressings, surgical gowns, and other healthcare textiles. The antimicrobial and UV-protective properties of turmeric-dyed silk could be further explored for controlled drug release systems and skin-contact therapeutics, leveraging chitosan’s biocompatibility and film-forming capabilities. Additionally, integrating turmeric’s antioxidant and anti-inflammatory properties into textile products offers potential applications in wearable therapeutics, merging the fields of functional textiles and pharmaceuticals to create next-generation bioactive materials.

## Figures and Tables

**Table 1:** Colouring, fastness, and air permeability of dyed silk samples.

Sample	Silk Picture	K/S	Colour Coordinates			Fastness				∆E*	Air Permeability (mm/s)
			L*	a*	b*	L	W	DR	WR		
T@Silk	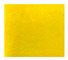	12.82	75.48	1.73	75.44	3–4	2–3	2–3	2–3	4.69	554.91
T-CS@Silk	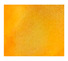	19.70	64.34	11.46	71.70	4–5	4–5	3–4	3–4	1.26	495.41
T-Al@Silk	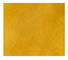	13.65	65.67	2.25	63.55	3–4	3–4	3–4	3–4	2.83	514.24

L: light, W: washing, DR: dry rubbing, WR: wet rubbing, ∆E*: colour difference in dyed silk pre- and post-washing.

**Table 2:** UV protection parameters of silk samples.

Silk Sample	UV Transmittance (%)		UV Blocking (%)		UPF	Ranking
	UV-A	UV-B	UV-A	UV-B		
Control	19.72	5.64	80.28	94.36	9	Poor
T@Silk	0.80	0.78	99.20	99.22	50^+^	Excellent
T-CS@Silk	0.73	0.68	99.27	99.32	50^+^	Excellent
T-Al@Silk	1.16	1.16	98.84	98.84	50^+^	Excellent

## Data Availability

Dataset available on request from the authors.
